# Supervised group Lasso with applications to microarray data analysis

**DOI:** 10.1186/1471-2105-8-60

**Published:** 2007-02-22

**Authors:** Shuangge Ma, Xiao Song, Jian Huang

**Affiliations:** 1 Department of Epidemiology and Public Health, Yale University, New Haven, CT 06520, USA; 2 Department of Health Administration, Biostatistics and Epidemiology, University of Georgia, Athens, GA 30602, USA; 3 Department of Statistics and Actuarial Science, University of Iowa, Iowa City, IA 52242, USA

## Abstract

**Background:**

A tremendous amount of efforts have been devoted to identifying genes for diagnosis and prognosis of diseases using microarray gene expression data. It has been demonstrated that gene expression data have cluster structure, where the clusters consist of co-regulated genes which tend to have coordinated functions. However, most available statistical methods for gene selection do not take into consideration the cluster structure.

**Results:**

We propose a supervised group Lasso approach that takes into account the cluster structure in gene expression data for gene selection and predictive model building. For gene expression data without biological cluster information, we first divide genes into clusters using the K-means approach and determine the optimal number of clusters using the Gap method. The supervised group Lasso consists of two steps. In the first step, we identify important genes within each cluster using the Lasso method. In the second step, we select important clusters using the group Lasso. Tuning parameters are determined using V-fold cross validation at both steps to allow for further flexibility. Prediction performance is evaluated using leave-one-out cross validation. We apply the proposed method to disease classification and survival analysis with microarray data.

**Conclusion:**

We analyze four microarray data sets using the proposed approach: two cancer data sets with binary cancer occurrence as outcomes and two lymphoma data sets with survival outcomes. The results show that the proposed approach is capable of identifying a small number of influential gene clusters and important genes within those clusters, and has better prediction performance than existing methods.

## Background

Development in microarray techniques makes it possible to profile gene expression on a whole genome scale and study associations between gene expression and occurrence or progression of common diseases such as cancer or heart disease. A large amount of efforts have been devoted to identifying genes that have influential effects on diseases. Such studies can lead to better understanding of the genetic causation of diseases and better predictive models. Analysis of microarray data is challenging because of the large number of genes surveyed and small sample sizes, and presence of cluster structure. Here the clusters are composed of co-regulated genes with coordinated functions. Without causing confusion, we use the phrases "clusters" and "gene groups" interchangeably in this article.

Available statistical approaches for gene selection and predictive model building can be roughly classified into two categories. The first type focuses on selection of individual genes. Examples of such studies range from early studies of detecting marginally differentially expressed genes under different experimental settings [1] to selecting important genes for prediction of binary disease occurrence [2,3] and detecting genes associated with patients' survival risks [4,5]. Since the dimension of gene expressions measured (~10^3–4^) is much larger than the sample size (~10^2^), variable selection or model reduction are usually needed. Previously employed approaches include the singular value decomposition [6], principal component analysis [7], partial least squares [3] and Lasso [8], among others. These approaches aim at identifying a small subset of genes or linear combinations of genes-often referred as super genes, that can best explain the phenotype variations. A limitation of these approaches is that the cluster structure of gene expression data is not taken into account.

Biologically speaking, complex diseases such as cancer, HIV and heart disease, are caused by mutations in gene pathways, instead of individual genes. Statistically speaking, there exist genes with highly correlated expressions and should be put into clusters [9]. Although functional groups and statistical clusters may not match perfectly, they tend to have certain correspondence [10,11].

The second type of methods focuses on detecting differential gene clusters. Examples include the global test [12], the maxmean approach [13] and the gene set enrichment analysis [14]. In classification and survival analysis, cluster-based approaches have also been considered [5,15]. One approach is to construct gene clusters first, which can be based on statistical measurements (for example K-means or Hierarchical methods) or biological knowledge [16] or both. Then the mean expression levels are used as covariates in downstream analysis [17]. With the simple cluster based methods, it is assumed that if a cluster is strongly associated with the outcome, then all genes within that cluster are associated with the outcome, which is not necessarily true. Within cluster gene selection may still be needed.

Lasso [18] is a popular method for variable selection with high-dimensional data, since it is capable of producing sparse models and is computationally feasible. For example, this method has been used for correlating survival with microarray data [8]. Standard Lasso approach carries out variable selection at the individual gene level. A recent development of the Lasso is the group Lasso method [19] (referred as GLasso hereafter). The GLasso is designed for selecting groups of covariates. In a recent study, [20] proposes logistic classification with the GLasso penalty and considers its applications in microarray study. Direct application of the GLasso can identify important gene groups. However, it is not capable of selecting important genes within the selected groups. The fitted model may not be sparse, especially if the clusters are large.

In this article, we propose a supervised group Lasso (SGLasso) approach, which selects both important gene clusters and important genes within clusters. Compared to individual gene based approaches such as Lasso, the SGLasso takes into consideration the cluster structure and can lead to better predictions, as shown in our empirical studies. Compared to cluster based methods such as the GLasso, the within-cluster gene selection aspect of SGLasso leads to more parsimonious models and hence more interpretable gene selection results. The proposed approach is applicable as long as the objective function is well defined and locally differentiable. In this article, we apply the SGLasso to logistic binary classification and Cox survival analysis problems with microarray gene expression data.

## Results

### Binary classification

#### Colon data

In this dataset, expression levels of 40 tumor and 22 normal colon tissues for 6500 human genes are measured using the Affymetrix gene chips. A selection of 2000 genes with the highest minimal intensity across the samples has been made by [2], and these data are publicly available at [21]. The colon data have been analyzed in several previous studies using other statistical approaches, see for example [3,22,23].

#### Nodal data

This dataset was first presented by [24,25]. It includes expression values of 7129 genes of 49 breast tumor samples. The expression data were obtained using the Affymetrix gene chip technology and are available at [26]. The response describes the lymph node (LN) status, which is an indicator for the metastatic spread of the tumor, a very important risk factor for the disease outcome. Among the 49 samples, 25 are positive (LN+) and 24 are negative (LN-). We threshold the raw data with a floor of 100 and a ceiling of 16000. Genes with max(*expression*)/min(*expression*) < 10 and/or max(*expression*) – min(*expression*) < 1000 are also excluded [1]. 3332 (46.7%) genes pass the first step screening. A base 2 logarithmic transformation is then applied. The Nodal data have also been studied by [22].

Although there is no limitation on the number of genes that can be used in the proposed approach, we first identify 500 genes for each dataset based on marginal significance to gain further stability as in [23]. Compute the sample standard errors of the *d *biomarkers *se*_(1)_,..., *se*_(*d*) _and denote their median as *med.se*. Compute the adjusted standard errors as 0.5(*se*_(1) _+ *med.se*),..., 0.5(*se*_(*d*) _+ *med.se*). Then the genes are ranked based on the *t*-statistics computed with the adjusted standard errors. The 500 genes with the largest absolute values of the adjusted *t*-statistics are used for classification. The adjusted *t*-statistic is similar to a simple shrinkage method discussed in [27].

**Figure 1:**
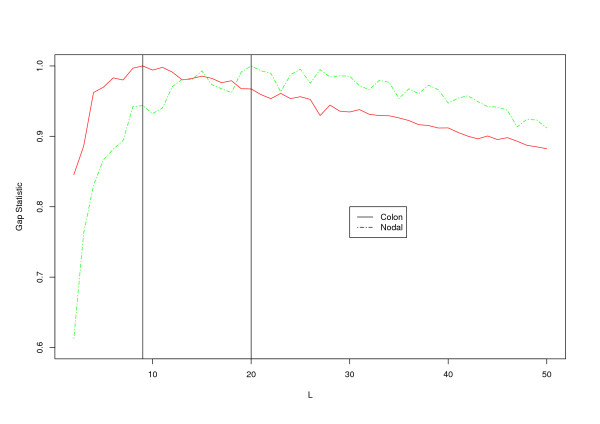
**Gap statistics as a function of number of clusters**. Red solid line: Colon data; Green dashed line: Nodal data.

For the Colon and Nodal data, clusters are constructed using the K-means approach and the Gap statistic is used to select the optimal number of clusters. We show in Figure 1 the Gap statistic as a function of the number of clusters. 9 clusters are constructed for the Colon data and 20 clusters are constructed for the Nodal data. Details of the clustering information are available upon request. With the generated clusters, we apply the proposed SGLasso approach. Tuning parameters are chosen using 3-fold cross validation. Summary model features are shown in Table 1. For the Colon data, 22 genes are present in the final model, representing 8 clusters. For the Nodal data, 66 genes are selected, representing 17 clusters. We list the identified genes in Tables 2 and 3.

**Table 1:** Comparison of estimation and prediction performance of different approaches.

		Lasso	Simple	GLasso	SGLasso
Colon	Nonzero	19	500	500	22
	Cluster	-	9	9	8
	Prediction	0.129	0.226	0.161	0.129

Nodal	Nonzero	37	500	233	66
	Cluster	-	20	9	17
	Prediction	0.245	0.163	0.122	0.122

Follicular	Nonzero	15	729	233	79
	Cluster	-	34	2	13
	Prediction	5.9	2.3	0.5	6.5

MCL	Nonzero	15	834	132	28
	Cluster	-	30	3	3
	Prediction	8.2	6.2	19.3	20.3

Nonzero: number of genes in the final models. Cluster: number of clusters in the final models. Prediction: for Colon and Nodal, Leave-One-Out prediction error; For Follicular and MCL, the logrank statistic.

**Table 2:** Colon data: genes with nonzero estimates from SGLasso.

Est.	Gene ID	Gene Description
0.229	Hsa.1047	Small Nuclear Ribonucleoprotein Associated Protein B/B';
0.385	Hsa.1410	TRANSLATIONAL INITIATION FACTOR 2 BETA SUBUNIT (HUMAN);
-0.058	Hsa.1039	Homo sapiens secretory pancreatic stone protein (PSP-S) mRNA

-0.110	Hsa.1013	PROFILIN I (HUMAN)
-0.018	Hsa.2809	IG MU CHAIN C REGION (HUMAN)
-0.072	Hsa.42949	ESTROGEN SULFOTRANSFERASE (Bos taurus)
-0.155	Hsa.1454	Human gamma amino butyric acid (GABAA) receptor beta-3 subunit mRNA

0.233	Hsa.8214	PUTATIVE SERINE/THREONINE-PROTEIN KINASE B0464.5 I
0.193	Hsa.1209	P14780 92 KD TYPE V COLLAGENASE PRECURSOR

-0.299	Hsa.8147	Human desmin gene, complete cds.
-0.511	Hsa.37937	MYOSIN HEAVY CHAIN, NONMUSCLE (Gallus gallus)

0.181	Hsa.462	Human serine kinase mRNA, complete cds.
0.484	Hsa.627	Human monocyte-derived neutrophil-activating protein (MONAP) mRNA
0.097	Hsa.601	Human aspartyl-tRNA synthetase alpha-2 subunit mRNA
-0.525	Hsa.696	Human cleavage stimulation factor

0.238	Hsa.1682	TRISTETRAPROLINE (HUMAN)
-0.492	Hsa.1832	MYOSIN REGULATORY LIGHT CHAIN 2, SMOOTH MUSCLE ISOFORM

-0.254	Hsa.612	Human beta adaptin mRNA
0.967	Hsa.6814	COLLAGEN ALPHA 2(XI) CHAIN (Homo sapiens)

0.189	Hsa.3306	Human gene for heterogeneous nuclear ribonucleoprotein core protein A1.
0.227	Hsa.3016	S-100P PROTEIN (HUMAN)
0.167	Hsa.2928	H.sapiens mRNA for p cadherin.

**Table 3:** Nodal data: genes with nonzero estimates from SGLasso.

Estimate	Gene ID	Gene Description
0.008	D63486_at	Human mRNA for KIAA0152 gene, complete cds
0.094	X74496_at	H.sapiens mRNA for prolyl oligopeptidase
0.014	Y10260_at	H.sapiens EYA1 gene

-0.063	U27185_at	Human RAR-responsive (TIG1) mRNA, complete cds
-0.062	U69263_at	Human matrilin-2 precursor mRNA, partial cds

0.001	D87673_at	Human mRNA for heat shock transcription factor 4, complete cds
-0.100	M83233_at	Homo sapiens transcription factor (HTF4A) mRNA, complete cds
0.011	U07223_at	Human beta2-chimaerin mRNA, complete cds
-0.121	X16354_at	Human mRNA for transmembrane carcinoembryonic antigen BGPa

0.451	M59916_at	Human acid sphingomyelinase (ASM) mRNA, complete cds
0.047	U88898_r_at	Human endogenous retroviral H protease
0.104	X97630_at	H.sapiens mRNA for serine/threonine protein kinase EMK

0.001	S83309_s_at	germ cell nuclear factor

-0.101	D87071_at	Human mRNA for KIAA0233 gene, complete cds
0.013	J00277_at	Human c-Ha-ras1 proto-oncogene, complete coding sequence
0.823	J02982_f_at	Human glycophorin B mRNA, complete cds
0.001	M69013_at	Human guanine nucleotide-binding regulatory protein mRNA
-0.096	X92396_at	H.sapiens mRNA for novel gene in Xq28 region
-0.091	Y00815_at	Human mRNA for LCA-homolog. LAR protein

-0.116	AB000114_at	Human mRNA for osteomodulin
0.016	D50532_at	Human mRNA for macrophage lectin 2, complete cds
-0.072	M83221_at	Homo sapiens I-Rel mRNA, complete cds
-0.061	X76717_at	H.sapiens MT-1l mRNA

0.031	J02645_at	Human translational initiation factor (eIF-2), alpha subunit mRNA
-0.070	X53587_at	Human mRNA for integrin beta 4

-1.323	AFFX-CreX-3_st	X03453 Bacteriophage P1 cre recombinase protein
-0.083	D80009_at	Human mRNA for KIAA0187 gene
0.019	J04615_at	Human lupus autoantigen mRNA, complete cds
0.009	L20861_at	Homo sapiens proto-oncogene (Wnt-5a) mRNA
-0.056	L20971_at	Human phosphodiesterase mRNA, complete cds
-0.026	M84820_s_at	Human retinoid X receptor beta (RXR-beta) mRNA, complete cds
0.156	U37408_at	Human CtBP mRNA, complete cds
0.079	U89336_cds7_at	receptor for advanced glycosylation end products gene
0.070	X76059_at	H.sapiens mRNA for YRRM1
-0.084	X82207_at	H.sapiens mRNA for beta-centractin (PC3)
0.025	X99687_at	H.sapiens mRNA for methyl-CpG-binding protein 2

0.501	L38933_rna1_at	the longest open reading frame predicts a protein of 202 amino acids

-0.034	U02493_at	Human 54 kDa protein mRNA, complete cds
0.016	U77846_rna1_s_at	Human elastin gene, Human elastin gene
-0.102	X07618_s_at	Human mRNA for cytochrome P450 db1 variant a
-0.614	X15357_at	Human mRNA for natriuretic peptide receptor (ANP-A receptor)
0.033	Y08265_s_at	H.sapiens mRNA for DAN26 protein, partial

-0.033	HG3521-HT3715_at	Ras-Related Protein Rap1b
-0.078	L33075_at	Homo sapiens ras GTPase-activating-like protein (IQGAP1) mRNA
0.011	X66364_at	H.sapiens mRNA PSSALRE for serine/threonine protein kinase

-0.094	AF009674_at	Homo sapiens axin (AXIN) mRNA, partial cds.
-0.379	AFFX-BioB-3_at	J04423 E coli bioB gene biotin synthetase
-0.184	AFFX-BioDn-3_at	J04423 E coli bioD gene dethiobiotin synthetase
0.058	HG2465-HT4871_at	Dna-Binding Protein Ap-2, Alt. Splice 3

0.017	D00762_at	Human mRNA for proteasome subunit HC8
-0.090	HG1612-HT1612_at	Macmarcks
-0.062	U09178_s_at	Human dihydropyrimidine dehydrogenase mRNA, complete cds
0.017	U29175_at	Human transcriptional activator (BRG1) mRNA, complete cds.
-1.184	U39817_at	Human Bloom syndrome protein (BLM) mRNA, complete cds
-0.211	U41344_at	Human prolargin (PRELP) gene, 5' flanking sequence
-0.080	X16832_at	Human mRNA for cathepsin H (EC 3.4.22.16)
0.013	X99226_at	H.sapiens mRNA for FAA protein
0.000	Z49878_at	H.sapiens mRNA for guanidinoacetate N-methyltransferase

-0.105	X68560_at	H.sapiens SPR-2 mRNA for GT box binding protein

0.048	HG3998-HT4268_at	L-Glycerol-3-Phosphate:Nad+ Oxidoreductase
0.001	U79285_at	Human clone 23828 mRNA sequence
0.003	X79981_at	H.sapiens VE-cadherin mRNA
0.024	X98176_at	H.sapiens mRNA for MACH-beta-1 protein.
0.001	Z18956_at	H.sapiens mRNA for taurine transporter

0.014	U18548_at	Human GPR12 G protein coupled-receptor gene, complete cds.
-0.281	Z22536_at	Homo sapiens ALK-4 mRNA, complete CDS

For the Colon data, gene Has.1039 has also been identified to be associated with Colon cancer in [28,29]. Gene Hsa.42949 is estrogen sulfotransferase. Research show that certain compounds, such as soy, have protective effect for colon cancer. The protective role of these compounds could be due to an ability to inhibit competitively the activation of promutagenic estrogen metabolites into carcinogens by estrogen sulfotransferases. The official symbol of gene Hsa.1454 is CSNK1E. Studies have revealed a negative regulatory function of CK1 in the Wnt signaling pathway, where CK1 acts as a negative regulator of the LEF-1/beta-catenin transcription complex, thereby protecting cells from development of cancer. Gene Hsa.8214 has official symbol DCGR6. It has been shown to be associated with mammary cancer and tumor cell proliferation in general. Gene Hsa.462 (official symbol SERPINC1) has been shown to be related to cancer cell proliferation. Gene Hsa.627 is also identified as a Colon cancer biomarker in [28]. Gene Hsa.696 has official symbol BTN1A1. RT-PCR analysis has revealed strongest expression of BTNL3 in small intestine, colon, testis, and leukocytes. Tristetraproline (gene Hsa.1682) has been reported to negatively regulate tumor necrosis factor alpha (TNF-alpha) production by binding the AU-rich element within the 3' noncoding sequences of TNF-alpha mRNA. Gene Hsa.3016 is S100P protein. 100P is expressed in human cancers, including breast, colon, prostate, and lung. In colon cancer cell lines, its expression level was correlated with resistance to chemotherapy.

For the Nodal data, gene U27185_at has official Symbol RARRES1. Also known as TIG1, the expression of this gene is upregulated by tazarotene as well as by retinoic acid receptors. Silencing of TIG1 promoter by hypermethylation is common in human cancers and may contribute to the loss of retinoic acid responsiveness in some neoplastic cells. The role of the matrilins (gene U69263) in tumorigenesis has not been studied. However, a related family of proteins (fibulins) has been implicated in cancer. Increased fibulin expression is seen in breast cancer, lung adenocarcinoma, colon cancer, and other solid tumors, suggesting that these proteins might play a role in tumor formation or progression. Gene U07223 is a member of the chimerin family and encodes a protein with a phorbol-ester/DAG-type zinc finger, a Rho-GAP domain and an SH2 domain. Decreased expression of this gene is associated with high-grade gliomas and breast tumors, and increased expression of this gene is associated with lymphomas. Findings suggest that CEACAM1 (gene X16354) participates in immune regulation in physiological conditions and in pathological conditions, such as inflammation, autoimmune disease, and cancer. Gene D87071_at is a confirmed breast cancer biomarker. Ras (gene J00277) and c-Myc play important roles in the up-regulation of nucleophosmin/B23 during proliferation of cells associated with a high degree of malignancy, thus outlining a signaling cascade involving these factors in the cancer cells. GNA11 (gene M69013) is involved in signaling of gonadotropin-releasing hormone receptor, which negatively regulates cell growth.

Down-regulation is suggested to be involved in human breast cancers. Gene D59532 encodes a member of the C-type lectin/C-type lectin-like domain (CTL/CTLD) superfamily. Members of this family share a common protein fold and have diverse functions, such as cell adhesion, cell-cell signaling, glycoprotein turnover, and roles in inflammation and immune response. Gene X53587 is human mRNA for integrin beta 4. Colonization of the lungs by human breast cancer cells is correlated with cell surface expression of the alpha(6)beta(4) integrin and adhesion to human CLCA2 (hCLCA2), Tumor cell adhesion to endothelial hCLCA2 is mediated by the beta(4) integrin, establishing for the first time a cell-cell adhesion property for this integrin that involves an entirely new adhesion partner. This adhesion is augmented by an increased surface expression of the alpha(6) beta(4) integrin in breast cancer cells selected in vivo for enhanced lung colonization but abolished by the specific cleavage of the beta(4) integrin with matrilysin. Wnt-5a (gene L20861) has been shown to influence the metastatic behavior of human breast cancer cells, and the loss of Wnt-5a expression is associated with metastatic disease. NFAT1, a transcription factor connected with breast cancer metastasis, is activated by Wnt-5a through a Ca2+ signaling pathway in human breast epithelial cells. Endogenous RXR beta (gene M84820) contributes to ERE binding activity in nuclear extracts of the human breast cancer cell line MCF-7. Detailed microscopic analysis of the morphology of MCF7 breast cancer cells lacking CtBPs (gene U37408) reveals an increase in the number of cells containing abnormal micronucleated cells and dividing cells with lagging chromosomes, indicative of aberrant mitotic chromosomal segregation. Methyl-CpG-binding domain protein-2 (gene X99687) mediates transcriptional repression associated with hypermethylated GSTP1 CpG islands in MCF-7 breast cancer cells. The nmt55/p54nrb protein (gene U02493) is post-transcriptionally regulated in human breast tumors leading to reduced expression in ER- tumors and the expression of an amino terminal altered isoform in a subset of ER+ tumors. Expression of elastin (gene U77846) in breast carcinoma cells has been demonstrated by immunohistochemistry and in situ hybridization. Cytochrome P450 1B1 (CYP1B1, gene X07618) is active in the metabolism of estrogens to reactive catechols and of different procarcinogens. The CYP1B1 gene polymorphisms do not influence breast cancer risk overall but may modify the risk after long-term menopausal hormone use. Genetic deficiency of DPYD enzyme (gene U09178) results in an error in pyrimidine metabolism associated with thymine-uraciluria and an increased risk of toxicity in cancer patients receiving 5-flourouracil chemotherapy.

We evaluate the prediction performance of the proposed approach via Leave-One-Out (LOO) cross validation. For the Colon and Nodal data, we compute the LOO cross validation errors. In this evaluation process, tuning parameters are computed using 3-fold cross validation for each reduced set. For comparison purposes, we also consider the following alternative approaches.

1. Lasso: we ignore the clustering structures and apply the Lasso directly. This approach has been considered in [18] for Cox survival analysis and [23] for logistic binary classification.

2. GLasso: we ignore the first step supervised selection and apply the GLasso directly. For binary classification, the GLasso has been investigated in [20].

3. Simple clustering: with the generated clusters, we compute the median of the gene expression level for each cluster. The medians are used as covariates. Since the number of "covariates" is less than the sample size, logistic/Cox models can be fit directly. This mimics the approach in [5].

For the alternative approaches, we also compute the LOO cross validation errors. Tuning parameters when presented are also chosen via 3-fold cross validation. Comparison results are shown in Table 1.

We can see from Table 1 that the SGLasso is capable of feature selection at both the cluster level and the within cluster gene level. The number of genes selected is much less than its counterpart from the simple clustering approach and GLasso. The Lasso is also capable of selecting a small number of genes. Especially we note that the number of genes selected by Lasso is smaller than by SGLasso. For simple data sets such as the Colon data, the Lasso prediction error is the same as the SGLasso. However for data sets that are more difficult to classify (Nodal), the SGLasso prediction error is much smaller. Both data sets have also been analyzed by other approaches. For the Colon data, ROC based approach has prediction error 0.14 [23]; LogitBoost has classification errors 0.145, 0.194 and 0.161 [22]; and classification tree has classification error 0.145 [22]. We note that since different sets of genes are used in those studies, Table 1 only provides rough comparisons. For the Nodal data, in [22], LogitBoost yields prediction error 0.184, 0.265 and 0.184, while classification tree has prediction error 0.204 and 1-nearest neighbor has prediction error 0.367.

### Survival analysis

#### Follicular lymphoma data

Follicular lymphoma is the second most common form of non-Hodgkin's lymphoma, accounting for about 22 percent of all cases. A study was conducted to determine whether the survival probability of patients with follicular lymphoma can be predicted by the gene-expression profiles of the tumors at diagnosis [5]. Fresh-frozen tumor-biopsy specimens and clinical data from 191 untreated patients who had received a diagnosis of follicular lymphoma between 1974 and 2001 were obtained. The median age at diagnosis was 51 years (range 23 to 81), and the median follow up time was 6.6 years (range less than 1.0 to 28.2). The median follow up time among patients alive at last follow up was 8.1 years. Eight records with missing survival information are excluded from the downstream analysis. Detailed experimental protocol can be found in [5].

Affymetrix U133A and U133B microarray genechips were used to measure gene expression levels from RNA samples. A log2 transformation was applied to the Affymetrix measurements. We first filter the 44928 gene measurements with the following criteria: (1) the max expression value of each gene across 191 samples must be greater than 9.186 (the median of the maximums of all probes). (2) the max-min should be greater than 3.874 (the median of the max-min of all probes). (3) Compute correlation coefficients of the uncensored survival times with gene expressions. Select the genes whose correlation with survival time is greater than 0.2. There are 729 genes that pass this screening process. We normalize genes across samples to have mean 0 and variance 1.

#### Mantel cell lymphoma data

[4] reported a study using microarray expression analysis of mantle cell lymphoma (MCL). The primary goal of this study was to discover genes that have good predictive power of patient's survival risk. Among 101 untreated patients with no history of previous lymphoma included in this study, 92 were classified as having MCL, based on established morphologic and immunophenotypic criteria. Survival times of 64 patients were available and other 28 patients were censored. The median survival time was 2.8 years (range 0.02 to 14.05 years). Lymphochip DNA microarrays [15] were used to quantify mRNA expression in the lymphoma samples from the 92 patients. The gene expression data that contain expression values of 8810 cDNA elements are available at [30].

We pre-process the data as follows to exclude noises and gain further stability: (1) Compute the variances of all gene expressions; (2) Compute correlation coefficients of the uncensored survival times with gene expressions; and (3) Select the genes with variances larger than the first quartile and with correlation coefficients larger than 0.25. 834 out of 8810 genes (16.5%) pass the above initial screening. We standardize these genes to have zero mean and unit variance.

We use the K-means method and Gap statistic in the cluster analysis. 34 (Follicular) and 30 (MCL) clusters are established. Plot similar to Figure 1 can be obtained and omitted here. Model estimation and prediction features are also provided in Table 1. We show in Tables 4 and 5 the genes included in the final models.

**Table 4:** Follicular data: genes with nonzero estimates from SGLasso.

Estimate	Gene ID	Gene Description
0.035	227117_at	CDNA FLJ40762 fis, clone TRACH2002847
-0.049	228671_at	hypothetical protein LOC199953
0.070	228776_at	gap junction protein, alpha 7, 45 kDa (connexin 45)
0.055	230448_at	hypothetical protein MGC15523
-0.042	230297_x_a	synaptic Ras GTPase activating protein 1 homolog
0.091	230938_x_a	activating transcription factor 5
0.053	209863_s_a	tumor protein p73-like

0.002	224125_at	pleckstrin homology domain containing, family N member 1
0.002	230826_at	monocyte to macrophage differentiation-associated 2
0.001	238605_at	Transcribed locus
0.005	222545_s_a	chromosome 10 open reading frame 57

0.062	239565_at	CDNA FLJ37010 fis, clone BRACE2009732
0.022	242904_x_a	
0.026	222015_at	Casein kinase 1, epsilon
0.032	219361_s_a	interferon stimulated exonuclease gene 20 kDa-like 1

0.046	223333_s_a	angiopoietin-like 4
0.084	224357_s_a	membrane-spanning 4-domains, subfamily A, member 4
0.046	204470_at	chemokine (C-X-C motif) ligand 1
0.023	205114_s_a	chemokine (C-C motif) ligand 3
0.118	208470_s_a	haptoglobin

0.058	237542_at	Transcribed locus
0.052	202953_at	complement component 1, q subcomponent, B chain
0.022	206214_at	phospholipase A2, group VII
0.085	210321_at	granzyme H (cathepsin G-like 2, protein h-CCPX)
0.054	214038_at	chemokine (C-C motif) ligand 8
-0.074	201841_s_a	heat shock 27 kDa protein 1
-0.028	211429_s_a	serpin peptidase inhibitor, clade A, member 1
-0.120	211470_s_a	sulfotransferase family, cytosolic, 1C, member 1
0.081	216950_s_a	Fc fragment of IgG, high affnity Ia, receptor (CD64)

-0.056	222694_at	hypothetical protein MGC2752
-0.042	232618_at	chromosome Y open reading frame 15A
-0.016	232874_at	Dedicator of cytokinesis 9
-0.034	237222_at	
0.024	240105_at	Chromosome 21 open reading frame 66
-0.016	241755_at	Ubiquinol-cytochrome c reductase core protein II
-0.032	242306_at	TPA regulated locus
-0.040	243705_at	DDHD domain containing 1

0.049	237131_at	hypothetical protein LOC645469
0.042	238359_at	
-0.045	242601_at	hypothetical protein LOC253012
0.049	243101_x_a	Chromosome 20 open reading frame 160
0.059	219360_s_a	transient receptor potential cation channel, subfamily M

-0.006	226665_at	AHA1, activator of heat shock 90 kDa protein ATPase homolog 2
-0.007	231852_at	
-0.009	241946_at	zinc finger, DHHC-type containing 21
-0.009	208067_x_a	ubiquitously transcribed tetratricopeptide repeat gene
-0.009	210973_s_a	fibroblast growth factor receptor 1
-0.006	220235_s_a	chromosome 1 open reading frame 103

-0.001	227697_at	suppressor of cytokine signaling 3
-0.006	227404_s_a	Early growth response 1
-0.004	235102_x_a	GRB2-related adaptor protein
-0.007	209189_at	v-fos FBJ murine osteosarcoma viral oncogene homolog
-0.001	213281_at	V-jun sarcoma virus 17 oncogene homolog (avian)
-0.008	201694_s_a	early growth response 1
-0.003	202672_s_a	activating transcription factor 3
0.002	AFFX-r2-Hs	

-0.088	223710_at	chemokine (C-C motif) ligand 26
-0.091	228844_at	solute carrier family 13, member 5
-0.023	233831_at	hypothetical protein LOC644752
-0.058	234062_at	CDNA FLJ12400 fis, clone MAMMA1002782
0.021	239574_at	Enoyl Coenzyme A hydratase domain containing 3
0.044	240142_at	
-0.140	215536_at	major histocompatibility complex, class II, DQ beta 2
-0.045	218935_at	EH-domain containing 3
-0.041	211177_s_a	thioredoxin reductase 2

0.014	231119_at	replication factor C (activator 1) 3, 38 kDa
0.032	232475_at	chromosome 15 open reading frame 42
0.022	237546_at	Transcribed locus
0.047	238201_at	
0.072	239670_at	
0.051	240607_at	Hypothetical protein LOC150271
0.039	241411_at	weakly similar to NP 055301.1 neuronal thread protein AD7c-NTP

0.002	223745_at	F-box protein 31
0.002	230280_at	tripartite motif-containing 9

-0.008	226771_at	ATPase, Class I, type 8B, member 2
-0.010	226869_at	Full length insert cDNA clone ZD77F06
-0.002	203029_s_a	protein tyrosine phosphatase, receptor type, N polypeptide 2
-0.005	209459_s_a	4-aminobutyrate aminotransferase
-0.009	221790_s_a	low density lipoprotein receptor adaptor protein 1

**Table 5:** MCL data: genes with nonzero estimates from SGLasso.

Estimate	Gene ID	Gene Description
0.011	24860	Hs.522568, Phosphatidylinositol-specific phospholipase C
0.005	26556	Hs.173438, Fas apoptotic inhibitory molecule
0.018	28537	Hs.120949, CD36 antigen
0.030	28640	Hs.84113, Cyclin-dependent kinase inhibitor 3
-0.004	28679	Hs.469723, RNA, U17D small nucleolar
0.005	30010	Hs.85137, Cyclin A2
0.009	32690	Hs.3104, Kinesin family member 14
0.010	32973	Hs.58992, SMC4 structural maintenance of chromosomes 4-like 1

0.078	27095	Hs.156346, Topoisomerase (DNA) II alpha 170 kDa
0.094	30157	Hs.497741, Centromere protein F, 350/400 ka
0.100	30898	Hs.532755, Likely ortholog of mouse gene trap locus 3
0.084	31049	Hs.241517, Polymerase (DNA directed), theta
0.080	34771	Hs.524390, Tubulin, alpha, ubiquitous

-0.067	16541	Hs.30054, Coagulation factor V
-0.065	23972	Hs.431009, Zinc finger protein, multitype 2
-0.036	24262	
-0.061	24379	Hs.120260, Immunoglobulin superfamily receptor translocation associated 1
-0.056	25058	Hs.298990, actin dependent regulator of chromatin
-0.101	25171	Hs.21388, Zinc finger, DHHC domain containing 21
-0.103	26192	Hs.530274, Aldolase B, fructose-bisphosphate
-0.037	27659	Hs.437336, Hypothetical protein MGC61571
-0.091	29653	Hs.40758, RAB30, member RAS oncogene family
-0.053	31196	Hs.508010, Fibronectin type III domain containing 3
-0.033	32497	
-0.076	32947	Hs.522863, Chromosome Y open reading frame 15A
-0.076	33506	Hs.364045, Hypothetical protein LOC92270
-0.059	33892	Hs.105956, Alpha 1,4-galactosyltransferase
-0.060	34438	Hs.368912, Dipeptidylpeptidase 4

For the Follicular data, gene 23098_x_a is associated with tumor protein p53. In transfected cells and KSHV-infected B lymphoma cells, KSHV-encoded latency-associated nuclear antigen (LANA) expression stimulates degradation of tumor suppressors von Hippel-Lindau and p53. In a recent case study with a Japanese girl who had EEC3 and developed diffuse large B-cell type non-Hodgkin lymphoma, researchers identified heterozygosity for a 1079A-G transition in exon 8 of the TP73L gene, resulting in a germline asp312-to-gly (D312G) mutation. Gene 223333_s_a is a member of the angiopoietin/angiopoietin-like gene family and encodes a glycosylated, secreted protein with a fibrinogen C-terminal domain. The encoded protein may play a role in several cancers and it also has been shown to prevent the metastatic process by inhibiting vascular activity as well as tumor cell motility and invasiveness. Gene 224357_s_a encodes a member of the membrane-spanning 4A gene family. Members of this nascent protein family are characterized by common structural features and similar intron/exon splice boundaries and display unique expression patterns among hematopoietic cells and nonlymphoid tissues. Chemokines (genes 204470_at, 205114_s_a) are a group of small (approximately 8 to 14 kD), mostly basic, structurally related molecules that regulate cell trafficking of various types of leukocytes through interactions with a subset of 7-transmembrane, G protein-coupled receptors. Chemokines also play fundamental roles in the development, homeostasis, and function of the immune system, and they have effects on cells of the central nervous system as well as on endothelial cells involved in angiogenesis or angiostasis. Serpin Al (gene 211429_s_a) has an invasion-promoting effect in anaplastic large cell lymphoma. Gene 216950_s_a has official symbol FCGR1A. Findings showed that both Fcgamma RIA and FcgammaRIIA mediated enhanced dengue virus immune complex infectivity but that FcgammaRIIA appeared to do so far more effectively. TRPM4-mediated (gene 219360_s_a) depolarization modulates Ca2+ oscillations, with downstream effects on cytokine production in T lymphocytes. The protein encoded by gene 210973_s_a is a member of the fibroblast growth factor receptor (FGFR) family, where amino acid sequence is highly conserved between members and throughout evolution. Chromosomal aberrations involving this gene are associated with stem cell myeloproliferative disorder and stem cell leukemia lymphoma syndrome. FGFR-1 is expressed in early hematopoietic/endothelial precursor cells, as well as in a subpool of endothelial cells in tumor vessels. Gene 227697_at encodes a member of the STAT-induced STAT inhibitor (SSI), also known as suppressor of cytokine signaling (SOCS), family. Over expression of suppressor of cytokine signaling 3 is associated with anaplastic large cell lymphoma.

Genes identified in the MCL study also have sound biological basis. When the positive cells are treated with phosphatidylinositol-specific phospholipase C (gene Hs.522568), a significant decrease in both stain intensity and percentage of positive cells is demonstrated by immunofluorescence. The protein encoded by gene Hs. 120949 is the fourth major glycoprotein of the platelet surface and serves as a receptor for thrombospondin in platelets and various cell lines. Since thrombospondins are widely distributed proteins involved in a variety of adhesive processes, this protein may have important functions as a cell adhesion molecule. Mutations in this gene cause platelet glycoprotein deficiency. The protein encoded by gene Hs.84113 belongs to the dual specificity protein phosphatase family. It was identified as a cyclin-dependent kinase inhibitor, and has been shown to interact with, and dephosphorylate CDK2 kinase, thus prevent the activation of CDK2 kinase. This gene was reported to be deleted, mutated, or overexpressed in several kinds of cancers. Studies show TOP2A (gene Hs.156346) is a proliferation marker, indicator of drug sensitivity, and prognostic factor in mantle cell lymphoma. This gene encodes a DNA topoisomerase, an enzyme that controls and alters the topologic states of DNA during transcription. The gene encoding this enzyme functions as the target for several anticancer agents and a variety of mutations in this gene have been associated with the development of drug resistance. Reduced activity of this enzyme may also play a role in ataxia-telangiectasia. Gene Hs.497741 encodes a protein that associates with the centromere-kinetochore complex. Autoantibodies against this protein have been found in patients with cancer or graft versus host disease. DNA Pol theta (gene Hs.241517) has a specialized function in lymphocytes and in tumor progression. Gene Hs.298990 encodes tumor suppressor proteins. The protein encoded by gene Hs. 105956 catalyzes the transfer of galactose to lactosylceramide to form globotriaosylceramide, which has been identified as the P(k) antigen of the P blood group system. PLG (gene Hs.368912) has the potential to simultaneously regulate calcium signaling pathways and regulate pHi via an association with NHE3 linked to DPP IV, necessary for tumor cell proliferation and invasiveness.

In the leave-one-out (LOO) based evaluation, denote β^
 MathType@MTEF@5@5@+=feaafiart1ev1aaatCvAUfKttLearuWrP9MDH5MBPbIqV92AaeXatLxBI9gBaebbnrfifHhDYfgasaacH8akY=wiFfYdH8Gipec8Eeeu0xXdbba9frFj0=OqFfea0dXdd9vqai=hGuQ8kuc9pgc9s8qqaq=dirpe0xb9q8qiLsFr0=vr0=vr0dc8meaabaqaciaacaGaaeqabaqabeGadaaakeaaiiWacuWFYoGygaqcaaaa@2E65@(-*i*) as the LOO estimate of *β *based on the reduced data set with the *i*^*th *^subject removed. We then compute the predicted risk score β^
 MathType@MTEF@5@5@+=feaafiart1ev1aaatCvAUfKttLearuWrP9MDH5MBPbIqV92AaeXatLxBI9gBaebbnrfifHhDYfgasaacH8akY=wiFfYdH8Gipec8Eeeu0xXdbba9frFj0=OqFfea0dXdd9vqai=hGuQ8kuc9pgc9s8qqaq=dirpe0xb9q8qiLsFr0=vr0=vr0dc8meaabaqaciaacaGaaeqabaqabeGadaaakeaaiiWacuWFYoGygaqcaaaa@2E65@(-*i*)Z′i
 MathType@MTEF@5@5@+=feaafiart1ev1aaatCvAUfKttLearuWrP9MDH5MBPbIqV92AaeXatLxBI9gBaebbnrfifHhDYfgasaacH8akY=wiFfYdH8Gipec8Eeeu0xXdbba9frFj0=OqFfea0dXdd9vqai=hGuQ8kuc9pgc9s8qqaq=dirpe0xb9q8qiLsFr0=vr0=vr0dc8meaabaqaciaacaGaaeqabaqabeGadaaakeaaieqacuWFAbGwgaqbamaaBaaaleaaieGacqGFPbqAaeqaaaaa@2F88@ for the *i*^*th *^subject. Since the Cox model is a special form of the transformation model, the uncensored survival time depends on ***β*Z**' via a generalized linear model. So the prediction evaluation can be based on comparing the survival functions of groups composed of different range of ***β*Z**'. A simple approach is to first dichotomize the predicted risk scores at the median to create two risk groups with equal sizes. We then compare the survival functions of the two generated risk groups. A significant difference (measured by the logrank test statistic with degree of freedom 1) indicates satisfactory prediction performance.

We show the prediction comparison in Table 1. We can again see that the models obtained under SGLasso are much smaller than those from the simple cluster approach and GLasso. However the SGLasso models are larger than their Lasso counterparts. For both data sets, the proposed SGLasso has the largest logrank statistics, indicating the best prediction performance. For the Follicular data, only Lasso and SGLasso have logrank statistics with p-value less than 0.05. The GLasso and simple approaches cannot properly predict survival based expressions. For the MCL data, all approaches have logrank statistics with p-value less than 0.05, with the largest logrank statistic from the SGLasso.

## Discussion

### Remark: clustering method selection

Gene expression clustering can be based on many approaches including the K-means, hierarchical, self-organizing map, and model based methods [31], among many others. Without making specific data assumptions, there do not exist optimal clustering method. The proposed K-means approach has been extensively used in microarray study. It is attractive because of its computational simplicity and optimality under the normal distribution assumption. We have also analyzed the four data sets using other clustering schemes including Hierarchical clustering. Our studies show that other approaches generate comparable or worse prediction results than the K-means approach. Since the K-means method yields satisfactory estimation and prediction results for the four data sets and other data (results not shown), we focus on the K-means approach only. A comprehensive comparison of different clustering is interesting but beyond the scope of this paper.

We propose using the Gap statistic for selecting the optimal number of clusters. Empirical studies in [32] and this paper show that it can lead to satisfactory results. We note again that there is no best selection method for number of clusters, unless stronger data assumptions are made. We refer to [33] for comprehensive discussions of gene clustering.

### Remark: prediction evaluation

In our examples, we carry out gene screening before analysis. The goal of such screening is to remove noisy genes and obtain more stable models. Gene screening has been employed in almost all microarray studies. We note that such screening may lead to bias in the prediction evaluation, since all records have been used in the screening. However since the number of genes passed screening is still large, the bias in the prediction is expected to be small. Especially all four approaches listed in Table 1 use the same sets of genes. So comparisons in Table 1 should be fair.

### Remark: two-step gene selection

The proposed SGLasso is a two-step approach. Another two-step gene selection approach is the supervised principal component analysis (SPCA, [34]). Significant differences exist between the SGLasso and other two-step approaches like SPCA. In other two-step approaches, the first supervised screening step considers all genes simultaneously. The cluster structure is ignored, whereas the main merit of the SGLasso is the usage of the cluster structure. Moreover, in SPCA, the selected features are the principal components. Although they may have satisfactory prediction performance, biological interpretations may not be clear. As a comparison, clear biological interpretations of gene identification results are available as shown in the Results section.

## Conclusion

Gene selection is essential in classification or survival analysis using high dimensional microarray data. Such selection can generate parsimonious, stable models with interpretable estimates. In this article, we propose the SGLasso approach. This approach explicitly takes into account the cluster structure and carries out feature selection at both the gene and cluster levels. Applications of this approach to four data sets show that it can produce parsimonious predictive models with satisfactory prediction performance.

Compared to available approaches, the SGLasso is the first to consider penalized gene selection at both the cluster level and the within cluster level. Compared to individual gene selection methods, the SGLasso is capable of taking cluster information into consideration. This makes it possible to reveal the associations between diseases and gene clusters. With the proposed approach, we can identify co-regulated genes which are jointly significantly associated with diseases. Compared to simple cluster based methods, SGLasso carries out the additional within cluster selection. This leads to a small number of genes within each cluster. So beyond identifying influential clusters, the proposed approach can also identify the genes that actually cause the association. From a scientific point of view, identifying important genes (beyond identifying important clusters) is critical.

As we point out, gene clusters can also be constructed based on biological information [16]. We should use such information whenever available. However we also note that such pathway information is far from complete or not available for many genes. In the absence of such information, we can use clustering methods to divide genes into groups. It is of interest to compare results based on statistical clustering with those based on biological clustering, when full pathway information is available. However, such empirical studies is beyond the scope of the current paper and will be pursued in later studies.

## Methods

### Gene clustering

The proposed SGLasso assumes that the cluster structure has been well defined. When clusters of genes in the same function groups can be constructed based on biological information such as GO [16], such clusters can be used in the analysis. However it is often the case that gene group information may only be partially available or even not available. In this case we propose defining cluster structure based on statistical measurements [9].

We use the K-means approach in this paper. There exist many alternative clustering methods, such as the hierarchical clustering, self-organizing map, tree-truncated vector quantization method, among others. For data sets with unknown data structures, there exists no dominating approach. We use the K-means approach since it is computationally affordable and relatively robust.

We propose using the Gap statistic [32] to determine the optimal number of clusters. With the K-means approach, we first choose *M*-the largest number of clusters. Then for *m *= 1,..., *M*:

1. Generate *m *clusters using the K-means approach. Denote *rss*_*m *_as the total within block sum of squares.

2. Create a new data set by separately permuting each gene expression measurements. Apply the K-means method to the permuted expression data. Let rss˜m
 MathType@MTEF@5@5@+=feaafiart1ev1aaatCvAUfKttLearuWrP9MDH5MBPbIqV92AaeXatLxBI9gBaebbnrfifHhDYfgasaacH8akY=wiFfYdH8Gipec8Eeeu0xXdbba9frFj0=OqFfea0dXdd9vqai=hGuQ8kuc9pgc9s8qqaq=dirpe0xb9q8qiLsFr0=vr0=vr0dc8meaabaqaciaacaGaaeqabaqabeGadaaakeaadaaiaaqaaiabdkhaYjabdohaZjabdohaZbGaay5adaWaaSbaaSqaaiabd2gaTbqabaaaaa@3348@ denote the resulting within cluster sum of squares. Repeat this for a number of times and compute the average *ave*(rss˜m
 MathType@MTEF@5@5@+=feaafiart1ev1aaatCvAUfKttLearuWrP9MDH5MBPbIqV92AaeXatLxBI9gBaebbnrfifHhDYfgasaacH8akY=wiFfYdH8Gipec8Eeeu0xXdbba9frFj0=OqFfea0dXdd9vqai=hGuQ8kuc9pgc9s8qqaq=dirpe0xb9q8qiLsFr0=vr0=vr0dc8meaabaqaciaacaGaaeqabaqabeGadaaakeaadaaiaaqaaiabdkhaYjabdohaZjabdohaZbGaay5adaWaaSbaaSqaaiabd2gaTbqabaaaaa@3348@).

3. Compute the Gap statistic as *gap*(*m*) = *ave*(rss˜m
 MathType@MTEF@5@5@+=feaafiart1ev1aaatCvAUfKttLearuWrP9MDH5MBPbIqV92AaeXatLxBI9gBaebbnrfifHhDYfgasaacH8akY=wiFfYdH8Gipec8Eeeu0xXdbba9frFj0=OqFfea0dXdd9vqai=hGuQ8kuc9pgc9s8qqaq=dirpe0xb9q8qiLsFr0=vr0=vr0dc8meaabaqaciaacaGaaeqabaqabeGadaaakeaadaaiaaqaaiabdkhaYjabdohaZjabdohaZbGaay5adaWaaSbaaSqaaiabd2gaTbqabaaaaa@3348@) - *rss*_*m*_.

Choose the value *m *that maximizes *gap*(*m*). We refer to [32] for detailed discussions of the Gap statistic.

### Data settings

Let **Z **be a length *d *vector of gene expressions, and let *Y *be the clinical outcome of interest. Assume that *n *i.i.d. copies of (*Y*, **Z**) are available. We generate *m *gene clusters using the K-means approach, where *m *is chosen using the Gap statistic. We assume that the clusters have sizes *p*_1_,..., *p*_*m *_with *p*_1 _+...+ *p*_*m *_= *d*. We denote **Z **= (**Z**^1^,...,**Z**^*m*^), where **Z**^i ^contains the *p*_*i *_gene expressions in the *i*^*th *^cluster for *i *= 1,..., *m*. We assume that *Y *is associated with **Z **through a parametric or semiparametric model *Y *~ *φ*(***β*Z**') with a regression function *φ *and unknown regression coefficient ***β***, where ***β ***= (***β***^1^,..., ***β***^*m*^) and ***β***^*i *^= (*β*^*i*1^,..., βipi
 MathType@MTEF@5@5@+=feaafiart1ev1aaatCvAUfKttLearuWrP9MDH5MBPbIqV92AaeXatLxBI9gBaebbnrfifHhDYfgasaacH8akY=wiFfYdH8Gipec8Eeeu0xXdbba9frFj0=OqFfea0dXdd9vqai=hGuQ8kuc9pgc9s8qqaq=dirpe0xb9q8qiLsFr0=vr0=vr0dc8meaabaqaciaacaGaaeqabaqabeGadaaakeaaiiGacqWFYoGydaahaaWcbeqaaiabdMgaPjabdchaWnaaBaaameaacqWGPbqAaeqaaaaaaaa@32CD@) for *i *= 1,..., *m*. In this article, we study the binary classification and censored survival analysis problems because of their wide applications.

### Binary classification

For classification problems, *Y *is the categorical variable indicating the disease status, for example occurrence or stage of cancer. We focus on binary classification only. Suppose that *Y *= 1 denotes the presence and *Y *= 0 indicates the absence of disease. We assume the commonly used logistic regression model, where the logit of the conditional probability is logit(*P*(*Y *= 1|**Z**)) = *α *+ ***β*Z**' and *α *is the unknown intercept. Based on a sample of *n *iid observations (*Y*_1_, **Z**_1_),..., (*Y*_*n*_, **Z**_*n*_), the maximum likelihood estimator is defined as (α˜
 MathType@MTEF@5@5@+=feaafiart1ev1aaatCvAUfKttLearuWrP9MDH5MBPbIqV92AaeXatLxBI9gBaebbnrfifHhDYfgasaacH8akY=wiFfYdH8Gipec8Eeeu0xXdbba9frFj0=OqFfea0dXdd9vqai=hGuQ8kuc9pgc9s8qqaq=dirpe0xb9q8qiLsFr0=vr0=vr0dc8meaabaqaciaacaGaaeqabaqabeGadaaakeaaiiGacuWFXoqygaacaaaa@2E61@, β^
 MathType@MTEF@5@5@+=feaafiart1ev1aaatCvAUfKttLearuWrP9MDH5MBPbIqV92AaeXatLxBI9gBaebbnrfifHhDYfgasaacH8akY=wiFfYdH8Gipec8Eeeu0xXdbba9frFj0=OqFfea0dXdd9vqai=hGuQ8kuc9pgc9s8qqaq=dirpe0xb9q8qiLsFr0=vr0=vr0dc8meaabaqaciaacaGaaeqabaqabeGadaaakeaaiiWacuWFYoGygaqcaaaa@2E65@) = argmax_*α*, *β *_*R*_*n *_(*α*, ***β***), where

Rn(α,β)=∑j=1nYjlog⁡(exp⁡(α+βZ′j)1+exp⁡(α+βZ′j))+(1−Yj)log⁡(11+exp⁡(α+βZ′j)).
 MathType@MTEF@5@5@+=feaafiart1ev1aaatCvAUfKttLearuWrP9MDH5MBPbIqV92AaeXatLxBI9gBaebbnrfifHhDYfgasaacH8akY=wiFfYdH8Gipec8Eeeu0xXdbba9frFj0=OqFfea0dXdd9vqai=hGuQ8kuc9pgc9s8qqaq=dirpe0xb9q8qiLsFr0=vr0=vr0dc8meaabaqaciaacaGaaeqabaqabeGadaaakeaacqWGsbGudaWgaaWcbaGaemOBa4gabeaakmaabmaabaacciGae8xSdeMaeiilaWcccmGae4NSdigacaGLOaGaayzkaaGaeyypa0ZaaabCaeaacqWGzbqwdaWgaaWcbaGaemOAaOgabeaaaeaacqWGQbGAcqGH9aqpcqaIXaqmaeaacqWGUbGBa0GaeyyeIuoakiGbcYgaSjabc+gaVjabcEgaNnaabmaabaWaaSaaaeaacyGGLbqzcqGG4baEcqGGWbaCdaqadaqaaiab=f7aHjabgUcaRiab+j7aIHqabiqb9PfaAzaafaWaaSbaaSqaaiabdQgaQbqabaaakiaawIcacaGLPaaaaeaacqaIXaqmcqGHRaWkcyGGLbqzcqGG4baEcqGGWbaCdaqadaqaaiab=f7aHjabgUcaRiab+j7aIjqb9PfaAzaafaWaaSbaaSqaaiabdQgaQbqabaaakiaawIcacaGLPaaaaaaacaGLOaGaayzkaaGaey4kaSYaaeWaaeaacqaIXaqmcqGHsislcqWGzbqwdaWgaaWcbaGaemOAaOgabeaaaOGaayjkaiaawMcaaiGbcYgaSjabc+gaVjabcEgaNnaabmaabaWaaSaaaeaacqaIXaqmaeaacqaIXaqmcqGHRaWkcyGGLbqzcqGG4baEcqGGWbaCdaqadaqaaiab=f7aHjabgUcaRiab+j7aIjqb9PfaAzaafaWaaSbaaSqaaiabdQgaQbqabaaakiaawIcacaGLPaaaaaaacaGLOaGaayzkaaGaeiOla4caaa@7DF2@

We always keep the intercept *α *in the model. For simplicity, we denote *R*_*n *_(*α*, ***β***) as *R*_*n *_(***β***).

### Survival analysis

For right censored survival data, *Y *= (*T*, Δ), where *T *= *min*(*U*, *V*) and Δ = *I*(*U *≤ *V*). Here *U *and *V *denote the event time of interest and the random censoring time, respectively. The most widely used model for right censored data is the Cox proportional hazards model [35] which assumes that the conditional hazard function *λ*(*u*|**Z**) = *λ*_0 _(*u*) exp(***β*Z**'), where *λ*_0 _is the unknown baseline function and ***β ***is the regression coefficient. Based on a sample of *n *iid observations *X*_*j *_= (*Y*_*j*_, **Z**_*j*_), *j *= 1,..., *n*, the maximum partial likelihood estimator is defined as the value β^
 MathType@MTEF@5@5@+=feaafiart1ev1aaatCvAUfKttLearuWrP9MDH5MBPbIqV92AaeXatLxBI9gBaebbnrfifHhDYfgasaacH8akY=wiFfYdH8Gipec8Eeeu0xXdbba9frFj0=OqFfea0dXdd9vqai=hGuQ8kuc9pgc9s8qqaq=dirpe0xb9q8qiLsFr0=vr0=vr0dc8meaabaqaciaacaGaaeqabaqabeGadaaakeaaiiWacuWFYoGygaqcaaaa@2E65@ that maximizes

Rn(β)=∏j=1n{exp⁡(βZ′j)∑k∈rjexp⁡(βZ′j)}δj,
 MathType@MTEF@5@5@+=feaafiart1ev1aaatCvAUfKttLearuWrP9MDH5MBPbIqV92AaeXatLxBI9gBaebbnrfifHhDYfgasaacH8akY=wiFfYdH8Gipec8Eeeu0xXdbba9frFj0=OqFfea0dXdd9vqai=hGuQ8kuc9pgc9s8qqaq=dirpe0xb9q8qiLsFr0=vr0=vr0dc8meaabaqaciaacaGaaeqabaqabeGadaaakeaacqWGsbGudaWgaaWcbaGaemOBa4gabeaakmaabmaabaaccmGae8NSdigacaGLOaGaayzkaaGaeyypa0ZaaebCaeaadaGadeqaamaalaaabaGagiyzauMaeiiEaGNaeiiCaa3aaeWaaeaaiiGacqGFYoGyieqacuqFAbGwgaqbamaaBaaaleaacqWGQbGAaeqaaaGccaGLOaGaayzkaaaabaWaaabeaeaacyGGLbqzcqGG4baEcqGGWbaCdaqadaqaaiab+j7aIjqb9PfaAzaafaWaaSbaaSqaaiabdQgaQbqabaaakiaawIcacaGLPaaaaSqaaiabdUgaRjabgIGiolabdkhaYnaaBaaameaacqWGQbGAaeqaaaWcbeqdcqGHris5aaaaaOGaay5Eaiaaw2haaaWcbaGaemOAaOMaeyypa0JaeGymaedabaGaemOBa4ganiabg+GivdGcdaahaaWcbeqaaiab+r7aKnaaBaaameaacqWGQbGAaeqaaaaakiabcYcaSaaa@5D65@

where *r*_*j *_= {*k *: *T*_*k *_≥ *T*_*j*_} is the risk set at time *T*_*j*_.

### Supervised group Lasso

For the logistic regression and Cox proportional hazards models, the SGLasso consists of the following steps.

1. For cluster *i *= 1,..., *m*, compute β^
 MathType@MTEF@5@5@+=feaafiart1ev1aaatCvAUfKttLearuWrP9MDH5MBPbIqV92AaeXatLxBI9gBaebbnrfifHhDYfgasaacH8akY=wiFfYdH8Gipec8Eeeu0xXdbba9frFj0=OqFfea0dXdd9vqai=hGuQ8kuc9pgc9s8qqaq=dirpe0xb9q8qiLsFr0=vr0=vr0dc8meaabaqaciaacaGaaeqabaqabeGadaaakeaaiiWacuWFYoGygaqcaaaa@2E65@^*i*^-the cluster-wise Lasso estimate of ***β***^*i*^. Especially,

β^
 MathType@MTEF@5@5@+=feaafiart1ev1aaatCvAUfKttLearuWrP9MDH5MBPbIqV92AaeXatLxBI9gBaebbnrfifHhDYfgasaacH8akY=wiFfYdH8Gipec8Eeeu0xXdbba9frFj0=OqFfea0dXdd9vqai=hGuQ8kuc9pgc9s8qqaq=dirpe0xb9q8qiLsFr0=vr0=vr0dc8meaabaqaciaacaGaaeqabaqabeGadaaakeaaiiWacuWFYoGygaqcaaaa@2E65@^*i *^= argmax *R*_*n *_(***β***^*i*^) subject to |*β*^*i*1^| + ... + |βipi
 MathType@MTEF@5@5@+=feaafiart1ev1aaatCvAUfKttLearuWrP9MDH5MBPbIqV92AaeXatLxBI9gBaebbnrfifHhDYfgasaacH8akY=wiFfYdH8Gipec8Eeeu0xXdbba9frFj0=OqFfea0dXdd9vqai=hGuQ8kuc9pgc9s8qqaq=dirpe0xb9q8qiLsFr0=vr0=vr0dc8meaabaqaciaacaGaaeqabaqabeGadaaakeaaiiGacqWFYoGydaahaaWcbeqaaiabdMgaPjabdchaWnaaBaaameaacqWGPbqAaeqaaaaaaaa@32CD@| ≤ *u*_*i*_,

where *u*_*i *_is the data-dependent tuning parameter and

Rn(βi)=∑j=1nYjlog⁡(exp⁡(α+βiZji′)1+exp⁡(α+βiZji′))+(1−Yj)log⁡(11+exp⁡(α+βiZji′))
 MathType@MTEF@5@5@+=feaafiart1ev1aaatCvAUfKttLearuWrP9MDH5MBPbIqV92AaeXatLxBI9gBaebbnrfifHhDYfgasaacH8akY=wiFfYdH8Gipec8Eeeu0xXdbba9frFj0=OqFfea0dXdd9vqai=hGuQ8kuc9pgc9s8qqaq=dirpe0xb9q8qiLsFr0=vr0=vr0dc8meaabaqaciaacaGaaeqabaqabeGadaaakeaacqWGsbGudaWgaaWcbaGaemOBa4gabeaakmaabmaabaaccmGae8NSdi2aaWbaaSqabeaacqWGPbqAaaaakiaawIcacaGLPaaacqGH9aqpdaaeWbqaaiabdMfaznaaBaaaleaacqWGQbGAaeqaaaqaaiabdQgaQjabg2da9iabigdaXaqaaiabd6gaUbqdcqGHris5aOGagiiBaWMaei4Ba8Maei4zaC2aaeWaaeaadaWcaaqaaiGbcwgaLjabcIha4jabcchaWnaabmaabaacciGae4xSdeMaey4kaSIae8NSdi2aaWbaaSqabeaacqWGPbqAaaacbeGccqqFAbGwdaqhaaWcbaGaemOAaOgabaGaemyAaKMccWaGqBOmGikaaaGaayjkaiaawMcaaaqaaiabigdaXiabgUcaRiGbcwgaLjabcIha4jabcchaWnaabmaabaGae4xSdeMaey4kaSIae8NSdi2aaWbaaSqabeaacqWGPbqAaaGccqqFAbGwdaqhaaWcbaGaemOAaOgabaGaemyAaKMccWaGqBOmGikaaaGaayjkaiaawMcaaaaaaiaawIcacaGLPaaacqGHRaWkdaqadaqaaiabigdaXiabgkHiTiabdMfaznaaBaaaleaacqWGQbGAaeqaaaGccaGLOaGaayzkaaGagiiBaWMaei4Ba8Maei4zaC2aaeWaaeaadaWcaaqaaiabigdaXaqaaiabigdaXiabgUcaRiGbcwgaLjabcIha4jabcchaWnaabmaabaGae4xSdeMaey4kaSIae8NSdi2aaWbaaSqabeaacqWGPbqAaaGccqqFAbGwdaqhaaWcbaGaemOAaOgabaGaemyAaKMccWaGqBOmGikaaaGaayjkaiaawMcaaaaaaiaawIcacaGLPaaaaaa@8D55@

for binary classification and

Rn(βi)=∏j=1n{exp⁡(βiZji′)∑k∈rjexp⁡(βiZji′)}δj
 MathType@MTEF@5@5@+=feaafiart1ev1aaatCvAUfKttLearuWrP9MDH5MBPbIqV92AaeXatLxBI9gBaebbnrfifHhDYfgasaacH8akY=wiFfYdH8Gipec8Eeeu0xXdbba9frFj0=OqFfea0dXdd9vqai=hGuQ8kuc9pgc9s8qqaq=dirpe0xb9q8qiLsFr0=vr0=vr0dc8meaabaqaciaacaGaaeqabaqabeGadaaakeaacqWGsbGudaWgaaWcbaGaemOBa4gabeaakmaabmaabaaccmGae8NSdi2aaWbaaSqabeaacqWGPbqAaaaakiaawIcacaGLPaaacqGH9aqpdaqeWbqaamaacmqabaWaaSaaaeaacyGGLbqzcqGG4baEcqGGWbaCdaqadaqaaiab=j7aInaaCaaaleqabaGaemyAaKgaaGqabOGae4NwaO1aa0baaSqaaiabdQgaQbqaaiabdMgaPPGamai0gkdiIcaaaiaawIcacaGLPaaaaeaadaaeqaqaaiGbcwgaLjabcIha4jabcchaWnaabmaabaGae8NSdi2aaWbaaSqabeaacqWGPbqAaaGccqGFAbGwdaqhaaWcbaGaemOAaOgabaGaemyAaKMccWaGqBOmGikaaaGaayjkaiaawMcaaaWcbaGaem4AaSMaeyicI4SaemOCai3aaSbaaWqaaiabdQgaQbqabaaaleqaniabggHiLdaaaaGccaGL7bGaayzFaaaaleaacqWGQbGAcqGH9aqpcqaIXaqmaeaacqWGUbGBa0Gaey4dIunakmaaCaaaleqabaacciGae0hTdq2aaSbaaWqaaiabdQgaQbqabaaaaaaa@6984@

for Cox survival analysis. That is for cluster *i*, we only use genes within that cluster to construct predictive models. Gene selection within that cluster is achieved with the Lasso. Sparse models are achieved when *u*_*i *_→ 0. We propose selection of *u*_*i *_using V-fold cross validation [36]. Especially we note that tuning parameters *u*_*i *_are selected for each cluster separately. So we allow different tuning parameters, hence different degrees of regularization for different clusters. This flexibility allows us to detect more subtle structures that cannot be detected by applying the Lasso method to all the genes/clusters at the same time.

2. For each cluster, the Lasso models have only a small number of nonzero coefficients. For cluster *i*, denote Z˜
 MathType@MTEF@5@5@+=feaafiart1ev1aaatCvAUfKttLearuWrP9MDH5MBPbIqV92AaeXatLxBI9gBaebbnrfifHhDYfgasaacH8akY=wiFfYdH8Gipec8Eeeu0xXdbba9frFj0=OqFfea0dXdd9vqai=hGuQ8kuc9pgc9s8qqaq=dirpe0xb9q8qiLsFr0=vr0=vr0dc8meaabaqaciaacaGaaeqabaqabeGadaaakeaaieqacuWFAbGwgaacaaaa@2DFE@^*i *^as the reduced covariate vector composed of covariates with nonzero estimated coefficients in Step 1 cluster-wise models. Denote β˜
 MathType@MTEF@5@5@+=feaafiart1ev1aaatCvAUfKttLearuWrP9MDH5MBPbIqV92AaeXatLxBI9gBaebbnrfifHhDYfgasaacH8akY=wiFfYdH8Gipec8Eeeu0xXdbba9frFj0=OqFfea0dXdd9vqai=hGuQ8kuc9pgc9s8qqaq=dirpe0xb9q8qiLsFr0=vr0=vr0dc8meaabaqaciaacaGaaeqabaqabeGadaaakeaaiiWacuWFYoGygaacaaaa@2E64@^*i *^as the corresponding reduced unknown coefficient. We note that the dimension of the genes may be greatly reduced via Step 1. For example in the examples, a cluster with size ~20 may only have 2 ~ 5 genes presented in the reduced data.

3. Construct the joint predictive model under the GLasso constraint. Especially,

β˜^=arg⁡max⁡R˜n(β˜) subject to |β˜1|+…+|β˜m|≤u,
 MathType@MTEF@5@5@+=feaafiart1ev1aaatCvAUfKttLearuWrP9MDH5MBPbIqV92AaeXatLxBI9gBaebbnrfifHhDYfgasaacH8akY=wiFfYdH8Gipec8Eeeu0xXdbba9frFj0=OqFfea0dXdd9vqai=hGuQ8kuc9pgc9s8qqaq=dirpe0xb9q8qiLsFr0=vr0=vr0dc8meaabaqaciaacaGaaeqabaqabeGadaaakeaaiiWacuWFYoGygaacgaqcaiabg2da9iGbcggaHjabckhaYjabcEgaNjGbc2gaTjabcggaHjabcIha4jqbdkfaszaaiaWaaSbaaSqaaiabd6gaUbqabaGcdaqadaqaaiqb=j7aIzaaiaaacaGLOaGaayzkaaGaeeiiaaIaee4CamNaeeyDauNaeeOyaiMaeeOAaOMaeeyzauMaee4yamMaeeiDaqNaeeiiaaIaeeiDaqNaee4Ba8MaeeiiaaYaaqWaaeaacuWFYoGygaacamaaCaaaleqabaGaeGymaedaaaGccaGLhWUaayjcSdGaey4kaSIaeSOjGSKaey4kaSYaaqWaaeaacuWFYoGygaacamaaCaaaleqabaGaemyBa0gaaaGccaGLhWUaayjcSdGaeyizImQaemyDauNaeiilaWcaaa@5FBB@

where R˜
 MathType@MTEF@5@5@+=feaafiart1ev1aaatCvAUfKttLearuWrP9MDH5MBPbIqV92AaeXatLxBI9gBaebbnrfifHhDYfgasaacH8akY=wiFfYdH8Gipec8Eeeu0xXdbba9frFj0=OqFfea0dXdd9vqai=hGuQ8kuc9pgc9s8qqaq=dirpe0xb9q8qiLsFr0=vr0=vr0dc8meaabaqaciaacaGaaeqabaqabeGadaaakeaacuWGsbGugaacaaaa@2DE8@_*n *_(β˜
 MathType@MTEF@5@5@+=feaafiart1ev1aaatCvAUfKttLearuWrP9MDH5MBPbIqV92AaeXatLxBI9gBaebbnrfifHhDYfgasaacH8akY=wiFfYdH8Gipec8Eeeu0xXdbba9frFj0=OqFfea0dXdd9vqai=hGuQ8kuc9pgc9s8qqaq=dirpe0xb9q8qiLsFr0=vr0=vr0dc8meaabaqaciaacaGaaeqabaqabeGadaaakeaaiiWacuWFYoGygaacaaaa@2E64@) is *R*_*n *_(***β***) with ***β ***replaced by β˜
 MathType@MTEF@5@5@+=feaafiart1ev1aaatCvAUfKttLearuWrP9MDH5MBPbIqV92AaeXatLxBI9gBaebbnrfifHhDYfgasaacH8akY=wiFfYdH8Gipec8Eeeu0xXdbba9frFj0=OqFfea0dXdd9vqai=hGuQ8kuc9pgc9s8qqaq=dirpe0xb9q8qiLsFr0=vr0=vr0dc8meaabaqaciaacaGaaeqabaqabeGadaaakeaaiiWacuWFYoGygaacaaaa@2E64@ and **Z **replaced by Z˜
 MathType@MTEF@5@5@+=feaafiart1ev1aaatCvAUfKttLearuWrP9MDH5MBPbIqV92AaeXatLxBI9gBaebbnrfifHhDYfgasaacH8akY=wiFfYdH8Gipec8Eeeu0xXdbba9frFj0=OqFfea0dXdd9vqai=hGuQ8kuc9pgc9s8qqaq=dirpe0xb9q8qiLsFr0=vr0=vr0dc8meaabaqaciaacaGaaeqabaqabeGadaaakeaaieqacuWFAbGwgaacaaaa@2DFE@. *u *is also chosen via V-fold cross validation. With *u *→ 0, estimates of some components of (β˜
 MathType@MTEF@5@5@+=feaafiart1ev1aaatCvAUfKttLearuWrP9MDH5MBPbIqV92AaeXatLxBI9gBaebbnrfifHhDYfgasaacH8akY=wiFfYdH8Gipec8Eeeu0xXdbba9frFj0=OqFfea0dXdd9vqai=hGuQ8kuc9pgc9s8qqaq=dirpe0xb9q8qiLsFr0=vr0=vr0dc8meaabaqaciaacaGaaeqabaqabeGadaaakeaaiiWacuWFYoGygaacaaaa@2E64@^1^,..., β˜
 MathType@MTEF@5@5@+=feaafiart1ev1aaatCvAUfKttLearuWrP9MDH5MBPbIqV92AaeXatLxBI9gBaebbnrfifHhDYfgasaacH8akY=wiFfYdH8Gipec8Eeeu0xXdbba9frFj0=OqFfea0dXdd9vqai=hGuQ8kuc9pgc9s8qqaq=dirpe0xb9q8qiLsFr0=vr0=vr0dc8meaabaqaciaacaGaaeqabaqabeGadaaakeaaiiWacuWFYoGygaacaaaa@2E64@^*m*^) can be exactly zero. Selection of important clusters can then be achieved.

In our examples, the objective functions *R*_*n *_are continuously differentiable and depend only on data and the unknown regression coefficient ***β***. Other smooth objective functions, for example the log-binomial likelihood for binary classification or the least square type estimating equation for the AFT survival model [37], can also be considered. The SGLasso only needs to assume that the expectation of the objective function has well-separated maximum. However for the proposed computational algorithms to work, we need to assume that the objective function is locally differentiable, i.e, it can be locally approximated by a smooth function.

### Computational algorithms

Since the Lasso constraint is not differentiable, standard derivative based maximization approaches, such as the Newton-Raphson, cannot be used to obtain the Lasso estimate. In most previous studies, the maximization relies on the quadratic programming (QP) or general non-linear programming which are known to be computationally intensive. Moreover, the quadratic programming cannot be applied directly to the settings where the sample size may be smaller than the number of predictors. The *L*_1_boosting based approach proposed by [38] provides a computationally feasible solution for high dimensional cases.

#### Algorithm I: *L*_1 _boosting Lasso

For the *i*^*th *^cluster:

1. Initialize ***β***^*i *^= 0 and *s *= 0.

2. With the current estimate of ***β***^*i *^= (*β*^*i*1^,..., βipi
 MathType@MTEF@5@5@+=feaafiart1ev1aaatCvAUfKttLearuWrP9MDH5MBPbIqV92AaeXatLxBI9gBaebbnrfifHhDYfgasaacH8akY=wiFfYdH8Gipec8Eeeu0xXdbba9frFj0=OqFfea0dXdd9vqai=hGuQ8kuc9pgc9s8qqaq=dirpe0xb9q8qiLsFr0=vr0=vr0dc8meaabaqaciaacaGaaeqabaqabeGadaaakeaaiiGacqWFYoGydaahaaWcbeqaaiabdMgaPjabdchaWnaaBaaameaacqWGPbqAaeqaaaaaaaa@32CD@), compute *φ *(***β***^*i*^) = *∂R*_*n *_(***β***^*i*^)/*∂****β***^*i*^. Denote the *k*^*th *^component of *φ *as *φ*^*k*^.

3. Find k* that minimizes min(*φ*^*k *^(***β***), - *φ*^*k *^(***β***)). If φk*
 MathType@MTEF@5@5@+=feaafiart1ev1aaatCvAUfKttLearuWrP9MDH5MBPbIqV92AaeXatLxBI9gBaebbnrfifHhDYfgasaacH8akY=wiFfYdH8Gipec8Eeeu0xXdbba9frFj0=OqFfea0dXdd9vqai=hGuQ8kuc9pgc9s8qqaq=dirpe0xb9q8qiLsFr0=vr0=vr0dc8meaabaqaciaacaGaaeqabaqabeGadaaakeaaiiGacqWFgpGzdaahaaWcbeqaaiabdUgaRnaaCaaameqabaGaeiOkaOcaaaaaaaa@3102@ (***β***) = 0, then stop the iteration.

4. Otherwise denote *γ *= -sign(φk*
 MathType@MTEF@5@5@+=feaafiart1ev1aaatCvAUfKttLearuWrP9MDH5MBPbIqV92AaeXatLxBI9gBaebbnrfifHhDYfgasaacH8akY=wiFfYdH8Gipec8Eeeu0xXdbba9frFj0=OqFfea0dXdd9vqai=hGuQ8kuc9pgc9s8qqaq=dirpe0xb9q8qiLsFr0=vr0=vr0dc8meaabaqaciaacaGaaeqabaqabeGadaaakeaaiiGacqWFgpGzdaahaaWcbeqaaiabdUgaRnaaCaaameqabaGaeiOkaOcaaaaaaaa@3102@ (***β***)). Find π^
 MathType@MTEF@5@5@+=feaafiart1ev1aaatCvAUfKttLearuWrP9MDH5MBPbIqV92AaeXatLxBI9gBaebbnrfifHhDYfgasaacH8akY=wiFfYdH8Gipec8Eeeu0xXdbba9frFj0=OqFfea0dXdd9vqai=hGuQ8kuc9pgc9s8qqaq=dirpe0xb9q8qiLsFr0=vr0=vr0dc8meaabaqaciaacaGaaeqabaqabeGadaaakeaaiiGacuWFapaCgaqcaaaa@2E80@ that

π^
 MathType@MTEF@5@5@+=feaafiart1ev1aaatCvAUfKttLearuWrP9MDH5MBPbIqV92AaeXatLxBI9gBaebbnrfifHhDYfgasaacH8akY=wiFfYdH8Gipec8Eeeu0xXdbba9frFj0=OqFfea0dXdd9vqai=hGuQ8kuc9pgc9s8qqaq=dirpe0xb9q8qiLsFr0=vr0=vr0dc8meaabaqaciaacaGaaeqabaqabeGadaaakeaaiiGacuWFapaCgaqcaaaa@2E80@ = argmax_*π *∈ [0,1] _*R*_*n *_((l - *π*) ***β ***+ *π u*_*i *_*γ*ηk*
 MathType@MTEF@5@5@+=feaafiart1ev1aaatCvAUfKttLearuWrP9MDH5MBPbIqV92AaeXatLxBI9gBaebbnrfifHhDYfgasaacH8akY=wiFfYdH8Gipec8Eeeu0xXdbba9frFj0=OqFfea0dXdd9vqai=hGuQ8kuc9pgc9s8qqaq=dirpe0xb9q8qiLsFr0=vr0=vr0dc8meaabaqaciaacaGaaeqabaqabeGadaaakeaaiiGacqWF3oaAdaahaaWcbeqaaiabdUgaRnaaCaaameqabaGaeiOkaOcaaaaaaaa@30F5@),

where ηk*
 MathType@MTEF@5@5@+=feaafiart1ev1aaatCvAUfKttLearuWrP9MDH5MBPbIqV92AaeXatLxBI9gBaebbnrfifHhDYfgasaacH8akY=wiFfYdH8Gipec8Eeeu0xXdbba9frFj0=OqFfea0dXdd9vqai=hGuQ8kuc9pgc9s8qqaq=dirpe0xb9q8qiLsFr0=vr0=vr0dc8meaabaqaciaacaGaaeqabaqabeGadaaakeaaiiGacqWF3oaAdaahaaWcbeqaaiabdUgaRnaaCaaameqabaGaeiOkaOcaaaaaaaa@30F5@ has the k*^th ^element equals to 1 and the rest equal to 0.

5. Let ***β***^*ik *^= (1 - π^
 MathType@MTEF@5@5@+=feaafiart1ev1aaatCvAUfKttLearuWrP9MDH5MBPbIqV92AaeXatLxBI9gBaebbnrfifHhDYfgasaacH8akY=wiFfYdH8Gipec8Eeeu0xXdbba9frFj0=OqFfea0dXdd9vqai=hGuQ8kuc9pgc9s8qqaq=dirpe0xb9q8qiLsFr0=vr0=vr0dc8meaabaqaciaacaGaaeqabaqabeGadaaakeaaiiGacuWFapaCgaqcaaaa@2E80@) ***β***^*ik *^for *k *≠ k* and βk*
 MathType@MTEF@5@5@+=feaafiart1ev1aaatCvAUfKttLearuWrP9MDH5MBPbIqV92AaeXatLxBI9gBaebbnrfifHhDYfgasaacH8akY=wiFfYdH8Gipec8Eeeu0xXdbba9frFj0=OqFfea0dXdd9vqai=hGuQ8kuc9pgc9s8qqaq=dirpe0xb9q8qiLsFr0=vr0=vr0dc8meaabaqaciaacaGaaeqabaqabeGadaaakeaaiiGacqWFYoGydaahaaWcbeqaaiabdUgaRnaaCaaameqabaGaeiOkaOcaaaaaaaa@30EA@ = (1 - π^
 MathType@MTEF@5@5@+=feaafiart1ev1aaatCvAUfKttLearuWrP9MDH5MBPbIqV92AaeXatLxBI9gBaebbnrfifHhDYfgasaacH8akY=wiFfYdH8Gipec8Eeeu0xXdbba9frFj0=OqFfea0dXdd9vqai=hGuQ8kuc9pgc9s8qqaq=dirpe0xb9q8qiLsFr0=vr0=vr0dc8meaabaqaciaacaGaaeqabaqabeGadaaakeaaiiGacuWFapaCgaqcaaaa@2E80@) βk*
 MathType@MTEF@5@5@+=feaafiart1ev1aaatCvAUfKttLearuWrP9MDH5MBPbIqV92AaeXatLxBI9gBaebbnrfifHhDYfgasaacH8akY=wiFfYdH8Gipec8Eeeu0xXdbba9frFj0=OqFfea0dXdd9vqai=hGuQ8kuc9pgc9s8qqaq=dirpe0xb9q8qiLsFr0=vr0=vr0dc8meaabaqaciaacaGaaeqabaqabeGadaaakeaaiiGacqWFYoGydaahaaWcbeqaaiabdUgaRnaaCaaameqabaGaeiOkaOcaaaaaaaa@30EA@ + *γu *π^
 MathType@MTEF@5@5@+=feaafiart1ev1aaatCvAUfKttLearuWrP9MDH5MBPbIqV92AaeXatLxBI9gBaebbnrfifHhDYfgasaacH8akY=wiFfYdH8Gipec8Eeeu0xXdbba9frFj0=OqFfea0dXdd9vqai=hGuQ8kuc9pgc9s8qqaq=dirpe0xb9q8qiLsFr0=vr0=vr0dc8meaabaqaciaacaGaaeqabaqabeGadaaakeaaiiGacuWFapaCgaqcaaaa@2E80@. Let *s *= *s *+ 1.

6. Repeat steps 2–5 until convergence or a fixed number of iterations *S *has been reached.

The ***β***^*i *^at convergence is the Lasso estimate. We conclude convergence if the absolute value of φk*
 MathType@MTEF@5@5@+=feaafiart1ev1aaatCvAUfKttLearuWrP9MDH5MBPbIqV92AaeXatLxBI9gBaebbnrfifHhDYfgasaacH8akY=wiFfYdH8Gipec8Eeeu0xXdbba9frFj0=OqFfea0dXdd9vqai=hGuQ8kuc9pgc9s8qqaq=dirpe0xb9q8qiLsFr0=vr0=vr0dc8meaabaqaciaacaGaaeqabaqabeGadaaakeaaiiGacqWFgpGzdaahaaWcbeqaaiabdUgaRnaaCaaameqabaGaeiOkaOcaaaaaaaa@3102@ (***β***) computed in step 3 is less than a pre-defined criteria, and/or if *R*_*n *_(***β***) is larger than a pre-defined threshold. Alternative algorithm can be LARS based. Since it is not the focus of this study, we omit discussions of other computational algorithms.

For the GLasso, a LARS based approach is proposed in [19]. With high dimensional cases, a computationally more affordable approach is proposed in [39]. This approach shares the same spirit as the *L*_1 _boosting Lasso and they are both special cases of the gradient-based constraint maximization discussed in [40]. This boosting based algorithm can be summarized into the following iterations.

#### Algorithm II: boosting group Lasso

1. Initialize β˜
 MathType@MTEF@5@5@+=feaafiart1ev1aaatCvAUfKttLearuWrP9MDH5MBPbIqV92AaeXatLxBI9gBaebbnrfifHhDYfgasaacH8akY=wiFfYdH8Gipec8Eeeu0xXdbba9frFj0=OqFfea0dXdd9vqai=hGuQ8kuc9pgc9s8qqaq=dirpe0xb9q8qiLsFr0=vr0=vr0dc8meaabaqaciaacaGaaeqabaqabeGadaaakeaaiiWacuWFYoGygaacaaaa@2E64@ = 0. Set Δ as a sufficiently small positive scalar.

2. With the current estimate of ***β***, calculate the gradient *∂R*_*n *_(β˜
 MathType@MTEF@5@5@+=feaafiart1ev1aaatCvAUfKttLearuWrP9MDH5MBPbIqV92AaeXatLxBI9gBaebbnrfifHhDYfgasaacH8akY=wiFfYdH8Gipec8Eeeu0xXdbba9frFj0=OqFfea0dXdd9vqai=hGuQ8kuc9pgc9s8qqaq=dirpe0xb9q8qiLsFr0=vr0=vr0dc8meaabaqaciaacaGaaeqabaqabeGadaaakeaaiiWacuWFYoGygaacaaaa@2E64@)/*∂*β˜
 MathType@MTEF@5@5@+=feaafiart1ev1aaatCvAUfKttLearuWrP9MDH5MBPbIqV92AaeXatLxBI9gBaebbnrfifHhDYfgasaacH8akY=wiFfYdH8Gipec8Eeeu0xXdbba9frFj0=OqFfea0dXdd9vqai=hGuQ8kuc9pgc9s8qqaq=dirpe0xb9q8qiLsFr0=vr0=vr0dc8meaabaqaciaacaGaaeqabaqabeGadaaakeaaiiWacuWFYoGygaacaaaa@2E64@.

3. Set *b *= β˜
 MathType@MTEF@5@5@+=feaafiart1ev1aaatCvAUfKttLearuWrP9MDH5MBPbIqV92AaeXatLxBI9gBaebbnrfifHhDYfgasaacH8akY=wiFfYdH8Gipec8Eeeu0xXdbba9frFj0=OqFfea0dXdd9vqai=hGuQ8kuc9pgc9s8qqaq=dirpe0xb9q8qiLsFr0=vr0=vr0dc8meaabaqaciaacaGaaeqabaqabeGadaaakeaaiiWacuWFYoGygaacaaaa@2E64@ - *∂*R˜
 MathType@MTEF@5@5@+=feaafiart1ev1aaatCvAUfKttLearuWrP9MDH5MBPbIqV92AaeXatLxBI9gBaebbnrfifHhDYfgasaacH8akY=wiFfYdH8Gipec8Eeeu0xXdbba9frFj0=OqFfea0dXdd9vqai=hGuQ8kuc9pgc9s8qqaq=dirpe0xb9q8qiLsFr0=vr0=vr0dc8meaabaqaciaacaGaaeqabaqabeGadaaakeaacuWGsbGugaacaaaa@2DE8@_*n *_(β˜
 MathType@MTEF@5@5@+=feaafiart1ev1aaatCvAUfKttLearuWrP9MDH5MBPbIqV92AaeXatLxBI9gBaebbnrfifHhDYfgasaacH8akY=wiFfYdH8Gipec8Eeeu0xXdbba9frFj0=OqFfea0dXdd9vqai=hGuQ8kuc9pgc9s8qqaq=dirpe0xb9q8qiLsFr0=vr0=vr0dc8meaabaqaciaacaGaaeqabaqabeGadaaakeaaiiWacuWFYoGygaacaaaa@2E64@)/*∂*β˜
 MathType@MTEF@5@5@+=feaafiart1ev1aaatCvAUfKttLearuWrP9MDH5MBPbIqV92AaeXatLxBI9gBaebbnrfifHhDYfgasaacH8akY=wiFfYdH8Gipec8Eeeu0xXdbba9frFj0=OqFfea0dXdd9vqai=hGuQ8kuc9pgc9s8qqaq=dirpe0xb9q8qiLsFr0=vr0=vr0dc8meaabaqaciaacaGaaeqabaqabeGadaaakeaaiiWacuWFYoGygaacaaaa@2E64@ and *τ *= {1,..., *m*}. Denote the *p*^*th *^component of *b *as *b*^*p*^.

4. Start Loop.

(a) Calculate the projection *u*_*p *_= *I *(*p *∈ *τ*) × (||*b*^*p*^|| + {*u *- ∑_*p*∈*τ *_||*b*^*p*^||}/|*τ*|) for *p *= 1,..., *m*, where *τ *is the cardinality of *τ*.

(b) If (*u*_*p *_≥ 0) for all *p*, then abort the loop.

(c) Else update the active set *τ *= {*p *: *u*_*p *_> 0}.

5. End Loop.

6. Get a new estimate β˜
 MathType@MTEF@5@5@+=feaafiart1ev1aaatCvAUfKttLearuWrP9MDH5MBPbIqV92AaeXatLxBI9gBaebbnrfifHhDYfgasaacH8akY=wiFfYdH8Gipec8Eeeu0xXdbba9frFj0=OqFfea0dXdd9vqai=hGuQ8kuc9pgc9s8qqaq=dirpe0xb9q8qiLsFr0=vr0=vr0dc8meaabaqaciaacaGaaeqabaqabeGadaaakeaaiiWacuWFYoGygaacaaaa@2E64@^*p *^= *b*^*p *^*u*_*p*_/||*b*^*p*^|| for *p *= 1,..., *m*.

7. Repeat steps 2–6 until convergence or a fixed number of iterations has been reached.

### A graphic presentation

We use the following numerical example to graphically demonstrate the parameter path of the proposed approach. For a better resolution, we only consider a small study with nine covariates (genes) and three clusters. Since the proposed approach does not depend on the special format of the objective function, we consider a simple linear regression model and use the least squares loss function.

Consider the linear model *y *= *β*_1 _*z*_1 _+ ... ... + *β*_9 _*z*_9 _+ *ε*, where *β *= (*β*_1_,..., *β*_9_) is the vector of regression coefficients and *ε *is the random error. We assume that there are three clusters, where (*z*_1_, *z*_2_, *z*_3_) form cluster 1, (*z*_4_, *z*_5_, *z*_6_) form cluster 2 and the rest belong to cluster 3. We assume that all *z *are marginally *N *(0, 1) distributed; the pairwise correlation coefficients are 0.4, 0.4 and 0.2 for covariates in clusters 1, 2, and 3, respectively; and different clusters are independent. Moreover, we set *β *= (-1, -1, 0, -1, -1, 0, 0, 0, 0). In this simulated dataset, we have three clusters, two of which are associated with the outcome. Within the first two clusters, two out of three covariates contribute to the outcome.

**Figure 2:**
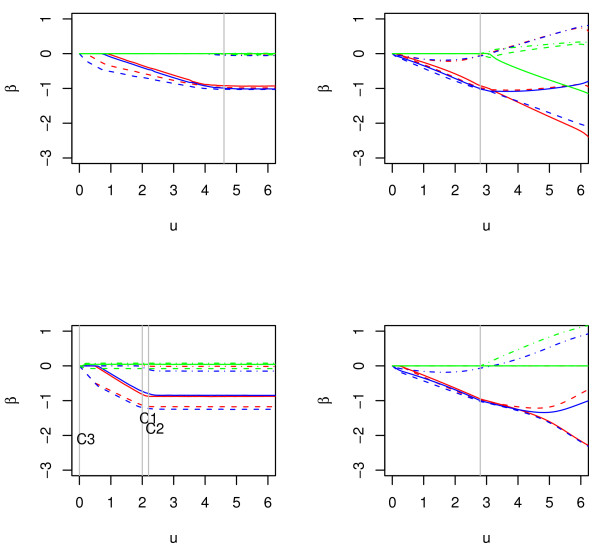
**Paths of parameter estimates for Lasso, GLasso and SGLasso**. Red lines, cluster 1; Blue lines, cluster 2; Green lines, cluster 3. Solid lines, *β*_1_, *β*_4 _and *β*_7_; Dashed lines, *β*_2_, *β*_5_, and *β*_8_; Dashed-Dotted lines, *β*_3_, *β*_6_, and *β*_9_. The grey lines show the selected tuning parameters. C1, C2 and C3 in the lower-left panel denote clusters 1, 2 and 3, respectively.

We generate 100 random data points from the above model. The regression parameters are estimated using the Lasso, GLasso and SGLasso. Tuning parameters are selected using 3-fold cross validation. In Figure 2, we show the parameter path as a function of the tuning parameter *u*. In the upper panels, we show the parameter paths for Lasso (left) and GLasso (right). In the lower-left panel, we show parameter paths for the first step estimates using the SGLasso. We see that the within-cluster Lasso has paths close to those in the upper-left panel. The parameter paths for the second step SGLasso (lower-right panel) are similar to those in the upper-right panel, with simpler structures due to the reduced number of covariates. The SGLasso selects (*z*_1_, *z*_2_, *z*_4_, *z*_5_, *z*_6_) with nonzero estimates, while the Lasso selects the covariates (*z*_1_,..., *z*_6_, *z*_8_), and the GLasso selects all covariates.

## Authors' contributions

SM designed the study and analyzed the data. XS designed the computational algorithms. JH contributed to the development of feature selection at the individual gene and cluster levels. All participated in writing or revising the paper.
